# The Immuno-Suppressive Effects of Cyclic, Environmental Heat Stress in Broiler Chickens: Local and Systemic Inflammatory Responses to an Intradermal Injection of Lipopolysaccharide

**DOI:** 10.3390/vetsci11010016

**Published:** 2023-12-30

**Authors:** Alessandro J. Rocchi, Jossie M. Santamaria, Chrysta N. Beck, Marites A. Sales, Billy M. Hargis, Guillermo Tellez-Isaias, Gisela F. Erf

**Affiliations:** Department of Poultry Science, Center of Excellence for Poultry Science, University of Arkansas System Division of Agriculture, Fayetteville, AR 72701, USA; ajrocch@clemson.edu (A.J.R.); jmsantam@uark.edu (J.M.S.); cnbeck@uark.edu (C.N.B.); marites.sales@usda.gov (M.A.S.); bhargis@uark.edu (B.M.H.); gtellez@uark.edu (G.T.-I.)

**Keywords:** inflammatory response, heat stress, chicken, leukocytes, reactive oxygen species, inflammatory cytokines, lipopolysaccharide, AGP-1

## Abstract

**Simple Summary:**

Heat stress is a growing concern in broiler production due to increasing environmental temperatures. Little is known about the effects of heat stress on the natural immune system defenses and health of broiler chickens. Using an environmental heat stress model in combination with minimally invasive tissue sampling techniques, the local and systemic, acute inflammatory responses to intradermal injection of bacterial component lipopolysaccharide or sterile saline (injection control) were examined in broilers reared in cyclic heat-stress- or thermoneutral-temperature conditions. This study revealed suppressive effects of heat stress on inflammatory responses initiated by lipopolysaccharide and by the sterile saline injection. Specifically, at the site of LPS injection, heat stressed broilers had lower infiltration of inflammatory leukocytes, reactive oxygen species generation, and expression of inflammatory factors than thermoneutral control broilers. Additionally, in the blood, concentrations of inflammatory leukocytes and plasma acute phase proteins did not increase in heat-stressed broilers like in thermoneutral broilers. Moreover, heat-stressed broilers had lower baseline concentrations of circulating lymphocytes than thermoneutral broilers. Hence, broilers reared in cyclic heat stress conditions have impaired ability to respond to bacterial challenges and associated tissue damage.

**Abstract:**

To assess effects of environmental heat stress (HS) on the local and systemic inflammatory responses to lipopolysaccharide (LPS), broilers were reared under thermoneutral (TN) or cyclic HS conditions. Thermoneutral temperatures followed commercial production settings, with HS broilers exposed to 35 °C for 14 h/day from 4 days onward. At 37 days, HS- and TN-broilers were assigned to either LPS (100 μg/mL) or endotoxin-free phosphate-buffered saline (PBS; vehicle) treatments, eight each to HS- and TN-LPS, four each to HS- and TN-PBS. Treatments were administered by intradermal injection of growing feather (GF) pulps; 10 μL/GF; 12 GF/broiler. Blood and GF were collected before and at 6 and 24 h post-injection to assess leukocyte population changes in GF-pulps and blood, reactive oxygen species (ROS) generation and cytokine expression in GF-pulps, and plasma concentrations of alpha-1 acid glycoprotein (AGP-1). HS-LPS broilers had lower (*p* ≤ 0.05) infiltration of heterophils and macrophages, ROS generation, and inflammatory cytokine expression in GF-pulps, and lacked the increases in heterophil, monocyte, and plasma AGP-1 concentrations observed in TN-LPS broilers. HS-broilers had similar or greater drops in blood lymphocytes 6 h post-LPS or -PBS injection, respectively, and lower baseline levels (*p* ≤ 0.05) of circulating T- and B-lymphocytes than TN-broilers. Results indicated that cyclic HS reduced the local and systemic acute inflammatory responses to LPS in broilers, likely impairing their innate defense against microbial infection.

## 1. Introduction

Environmental heat stress is a growing concern within the poultry industry. Broilers subjected to environmental heat stress were shown to experience hyperthermia along with a reduction in feed intake, nutrient absorption, and growth rate, as well as an increase in water intake, morbidity, and mortality [1,2,3,4]. The intestinal dysfunction associated with environmental heat stress involves oxidative stress, immune injury, and a weakening of the gut barriers. Particularly, the disruption of tight junction proteins within the gut increases permeability and allows for paracellular translocation of toxins and bacteria inside the body [3,4,5,6,7]. Moreover, reduced secretory IgA content and number of epithelial lymphocytes in the duodenal, jejunal, and ileal mucosa further contribute to the reduction in immune protection at the gut barrier in broilers subjected to heat stress [8,9,10]. The physiological stress placed upon the chickens by environmental heat stress may also account for the observed negative effects of heat stress on immune system development and function. Specifically, systemic effects of environmental heat stress on the chicken’s immune system include reductions in relative weights of primary and secondary lymphoid organs, antibody responses to SRBC, concentrations of IgA and cytokines in the blood, and innate immune activities, as indicated by differential protein expression profiles in spleens [11,12,13,14].

Although general suppression of the chicken’s immune system by environmental heat stress has been identified, there is a current lack of information concerning heat-stress effects on the inflammatory response to microbial infections. In general terms, the inflammatory response is defined as the recruitment of leukocytes, plasma proteins, and fluids from the blood to the affected tissue. Inflammatory responses that quickly recruit large numbers of appropriate leukocytes, activate antimicrobial activities, eliminate or contain the infection, and reestablish homeostasis, constitute a critically important mechanism of the innate immune system [15,16,17].

Lipopolysaccharide (LPS), a cell wall component of Gram-negative bacteria and a potent inducer of an inflammatory response, is commonly used to evaluate innate immune response capabilities. In chickens, a one-time administration of LPS into a complex tissue, like the dermis of the skin, will typically result in vascular changes facilitating the rapid recruitment of heterophils from the blood. The influx of heterophils is accompanied and followed by monocyte/macrophage infiltration. The recruited leukocytes are stimulated to carry out various antimicrobial activities, including generating reactive oxygen species, expressing inflammatory cytokines and chemokines, activating enzymes, and producing various bactericidal substances. The local acute inflammatory response initiated by LPS is also associated with changes in peripheral blood cell and protein profiles [18,19,20,21,22].

French et al. [23] recently reported on a “two-window approach” to study the local and systemic acute inflammatory responses to intradermal (i.d.) injection of LPS in individual broilers [23]. This approach involved minimally invasive techniques, allowing for repeated and concurrent sampling of an individual’s tissue and peripheral blood for ex vivo analyses. Specifically, in French et al. [23], LPS was i.d. injected into the pulp of multiple growing feathers (GF) on each broiler [24,25]. GF and blood were collected before (0 h) and at 6 and 24 h post-GF-pulp injection. Laboratory analyses of collected samples revealed extensive recruitment of heterophils and monocytes/macrophages into the dermis of injected GF, reaching peak levels at 6 and 24 h post-injection, respectively. Local cellular activities in GF-pulps included the generation of reactive oxygen species (ROS), mRNA expression of inflammatory cytokines (e.g., interleukin-1 (IL-1), IL-6, IL-8), and antioxidant enzyme activity. In the blood, the concentrations of heterophils were elevated at 6 h and returned to baseline levels by 24 h, whereas lymphocyte concentrations dropped at 6 h and returned to pre-injection levels by 24 h [23]. Overall, this model has proven effective in monitoring local and systemic inflammatory responses to microbes.

The objective of the current study was to examine the effects of cyclic, environmental heat stress on the local (GF-pulp) and systemic (blood) acute inflammatory responses to LPS injected into the dermis of GF-pulps in broilers reared at thermoneutral (TN) or cyclic heat stress (HS) temperatures. For TN-broilers, TN conditions followed step-down, standard industry temperature settings. From Day 4 onward, HS-broilers were subjected to cyclic environmental heat stress simulating hot summer days, with 35 °C for 14 h and TN-temperatures for the remaining hours of each day. The inflammatory response was induced by i.d. GF-pulp injections of LPS when the broilers were 37 days of age. GF and blood were collected before (0 h) and at 6 and 24 h post-injection for laboratory analyses. Aspects examined in GF-pulps included relative amounts and types of leukocyte populations present, ROS generation, and relative cytokine mRNA expression, and in blood, concentrations of various cell populations and plasma alpha-1 glycoprotein (AGP-1), an acute phase protein. The responses to i.d. GF-pulp injection of endotoxin-free PBS (vehicle injection control) were also examined in TN- and HS-broilers.

## 2. Materials and Methods

### 2.1. Experimental Animals and Rearing Conditions

Newly hatched Cobb 500 broiler chicks were tagged at hatch. Based on tag number, the chicks were then randomly assigned to eight chambers of the University of Arkansas System, Division of Agriculture’s (UADA) Poultry Environmental Research Laboratory (PERL) in Fayetteville, Arkansas. The eight environmental chambers were then randomly assigned to TN (4) or HS (4) temperature conditions, and each chamber was evenly split into two pens to produce eight pens per temperature treatment (16 pens total). Twenty-three birds were placed into each pen, on wood shavings with 10 birds/m^2^ stocking density. All protocols and procedures involving animals used in this trial were approved by the UA Institutional Animal Care and Use Committee (IACUC protocol #21018).

From Day 0 to 3, all chicks were reared at 32 °C. A standard industry step-down procedure was followed for TN temperature conditions, i.e., Day 4 to 6, 31 °C; Day 7 to 10, 29 °C; Day 11–14, 26 °C; and Day 15 onwards, 24 °C. From Day 4 onwards, HS chicks were subjected daily to 35 °C from 8 am to 10 pm (HS temperature; 14 h) and to TN temperature from 10 pm to 8 am (10 h), while TN chicks were kept at TN temperatures 24 h a day. For all treatment groups, diets and lighting schedules followed industry standards for broilers (i.e., Starter diet Day 0 to 10, Grower diet Day 11 to 28, and Finisher diet Day 28–42; 24 h of light Day 0 to 1; 23 h of light with 1 h of dark Day 2 to 7; 20 h of light with 4 h of dark Day 8 to 14; and 18 h of light with 6 h of dark Day 15 to 42).

### 2.2. Experimental Induction of the Inflammatory Response

At 18 days of age, four treatment groups were formed based on future GF-pulp injection of either *Salmonella typhimurium* lipopolysaccharide (LPS; Sigma Chemical Company Saint Louis, MO, USA) prepared in endotoxin-free Dulbecco’s phosphate-buffered saline (PBS; Sigma, catalog number L6511) or PBS vehicle, and either TN or HS temperature conditions (i.e., TN-LPS, HS-LPS, TN-PBS and HS-PBS). Three broilers were randomly selected from each chamber, two for LPS (one per pen) and one for PBS (vehicle) injection, resulting in eight birds for each TN-LPS and HS-LPS, and four birds for each TN-PBS and HS-PBS treatment groups. A row of eight GFs were then plucked from each breast tract of the broilers to yield uniform, 19-day-old, regenerated GFs for injection when the broilers were 37 days of age. On Day 37, the pulp of 6 regenerated GFs from each breast tract (12 GF total/broiler) of TN-LPS and HS-LPS broilers were injected with 10 μL LPS (100 μg/mL; 1 μg/GF; 12 μg per broiler) or 10 μL of PBS for the LPS and PBS treatment groups, respectively, as described in [26].

### 2.3. Blood and Growing Feather (GF) Sample Collections

One mL of heparinized blood and six GFs were collected from each bird before (0 h) and at 6 and 24 h post-intradermal (i.d.) GF-pulp injection. At each time-point, two of the collected GF were used to prepare pulp cell suspensions, while four GFs were snap-frozen in liquid nitrogen and stored at −80 °C. Blood was used to prepare blood smears and diluted blood cell suspensions, and to isolate plasma. Plasma was stored at −80 °C [26].

### 2.4. Preparation of GF Pulp- and Blood-Cell Suspensions

Pulp cell suspensions were prepared as described previously [23,25]. Briefly, for each bird and time point, the entire pulp of two GFs was pulled out of the sheath and placed in 1 mL PBS containing 0.1% collagenase type IV (Life Technologies, Carlsbad, CA, USA) and 0.1% dispase II (Boehringer Mannheim, Mannheim, Germany). After a 10 min incubation at 37 °C, the pulps were gently pushed through a 60 μm nylon mesh while continuously adding ice-cold PBS. The pulp cell suspensions were washed twice in PBS at 250× *g* for 4 min at 4 °C, and the pellet resuspended to a final volume of 0.5 mL with PBS. Cell suspensions were kept on ice until use for immunofluorescent staining or determination of ROS generation by kinetic fluorescence assay.

Heparinized blood was diluted 50-fold by adding 20 μL blood to 980 μL of PBS+ staining buffer (PBS, 1% bovine serum albumin, 0.1% sodium azide) [27].

### 2.5. Immunofluorescent Staining and GF-Pulp- and Blood-Cell Population Analyses

Leukocyte populations in the GF-pulp and blood-cell suspensions were identified using direct, two- and three-color, immunofluorescent staining procedures, as described in [23,25]. A cocktail of mouse monoclonal antibodies (mAbs) to chicken CD41/61 conjugated to fluorescein isothiocyanate (FITC), KUL01 conjugated with phycoerythrin (PE), and CD45 conjugated to spectral red (SPRD) was used to identify thrombocytes, monocytes/macrophages, and total leukocytes, respectively. Another cocktail consisted of Bu-1-PE and CD3-SPRD mAbs to identify chicken B- and T-lymphocytes, respectively. Heterophils were identified based on size and internal complexity characteristics (forward and side scatter, respectively) of CD45^+^ pulp and blood leukocytes, whereby for blood, total leukocytes were defined as CD45^+^CD41/61^−^ cells as thrombocytes express low levels of CD45 [23,27]. Controls were included to detect non-specific binding of fluorescently labeled mAbs, determine cut-offs between positive and negative fluorescence, and to set compensations [23]. Except for the CD41/61-FITC mAb purchased from Bio-Rad, Hercules, CA, USA, all mAbs were purchased from Southern Biotech, Birmingham, AL, USA. All cell samples were analyzed by flow cytometry using a BD C6-plus Accuri flow cytometer (Becton Dickinson, San Jose, CA, USA). Pulp data were expressed as the percentage of stained cells in the total pulp cell suspension, whereas for blood, cell concentrations (cells/μL) were reported. For both pulp and blood, lymphocyte data were calculated as the sum of T and B cells. For blood the heterophil to lymphocyte (H:L) ratios were calculated by dividing the concentrations of heterophils by the concentrations of lymphocytes. The proportions of blood basophils and eosinophils were determined by microscopic evaluation of at least 300 white blood cells (WBC) on Wright-stained blood smears prepared for each blood sample. The concentrations of basophils and eosinophils were then calculated by multiplying the concentration of WBC (CD45^+^CD41/61^−^) determined by flow cytometry by the percentage of basophils or eosinophils and dividing the products by 100.

### 2.6. Reactive Oxygen Species Generation Assay

Reactive oxygen species generation in pulp cell suspensions was determined for each pulp cell suspension by kinetic fluorescence assay using 2′,7′-dichlorofluorescin-diacetate (DCFDA, Sigma, St. Louis, MO, USA), as described in [28]. Generation of fluorescence from the samples was determined via kinetic read for 1.0 h at 10 min intervals using a fluorescence microplate reader (Synergy HTX; BioTek, Winooski, VT, USA) set at 37 °C with an excitation wavelength of 485 nm and an emission wavelength of 530 nm. Data were acquired with Gen5 software 2.07 (BioTek). Appropriate controls for autofluorescence and background fluorescence were included in each assay. A line of best fit describing the relationship between fluorescence (a.u.) and time was calculated for each sample and the relative amount of ROS generation reported as the slope of the best fit equation [23].

### 2.7. Isolation of RNA from GF-Pulps and Relative Gene Expression Analyses of Cytokines by Quantitative Reverse Transcription PCR

For each bird and time point, pulps were extracted from two frozen GFs and total RNA isolated, as described in [23]. Total RNA quality and concentration were determined using NanoDrop (Thermo Fisher Scientific, Waltham, MA, USA). RNA was transcribed to cDNA using the High-Capacity cDNA kit with MultiScribe Reverse Transcriptase following the manufacturer’s protocol (Thermo Fisher Scientific). All cDNA samples were diluted to a working concentration of 10 ng/μL with nuclease-free water and stored at −20 °C until used for relative gene expression analyses [23].

Primers and probes for interleukin 1β (*IL1β*), *IL6*, *IL8* (*CXCL8*), tumor-necrosis factor-α (*TNFα*), *IL10*, and transforming growth factor-β-1 (*TGFβ1*) and the 28S reference gene, as well as the qPCR procedure were as described [23]. Real-time qPCR was performed using TaqMan Universal Master Mix with UNG protocol in a 7500 Fast Real-Time PCR System (Thermo Fisher Scientific), as described in [23]. Using 28S as the reference gene to calculate target gene delta Ct, the relative gene expression was determined as 40 minus delta Ct [29].

### 2.8. Plasma Alpha-1 Acid Glycoprotein-1 Assay

Alpha-1 acid glycoprotein (AGP-1) concentrations (mg/mL) in plasma of blood samples collected before (0 h) and at 6 and 24 h following i.d. GF-pulp injection of lipopolysaccharide (LPS) or diluent (PBS) were determined by chicken AGP-1 ELISA following manufacturer’s protocol (Abcam, Waltham, MA, USA).

### 2.9. Statistical Analysis

Statistical analyses were conducted using Sigma Plot 13 Statistical Software (Systat Software, Inc., San Jose, CA). Three-way analysis of variance (ANOVA) was conducted to determine the effects of temperature (TN, HS), time (0, 6, 24 h), and injection (LPS, PBS) and their interactions. Significant interactions involving injection treatment were revealed. Hence, data for LPS and PBS injection were separately analyzed by 2-way ANOVA for GF-pulp data, or 2-way repeated measures (RM) ANOVA for blood data, to determine the effects of temperature and time, and temperature by time interactions. Shapiro–Wilk Normality test and Brown–Forsythe Equal variance tests were conducted for all ANOVAs; in the few cases when normality and equal variance tests failed (*p* ≤ 0.05), log transformation of data restored normality and equal variance (*p* > 0.05). In the presence of significant main effects, Holm–Sidak Pairwise Multiple Comparison Procedures were conducted as appropriate. In all cases, statistical significance was considered *p* ≤ 0.05.

## 3. Results

### 3.1. Leukocyte Profiles in GF-Pulps and Blood before and after i.d. GF-Pulp Injection of LPS or PBS

#### 3.1.1. Heterophils

**Figure 1:**
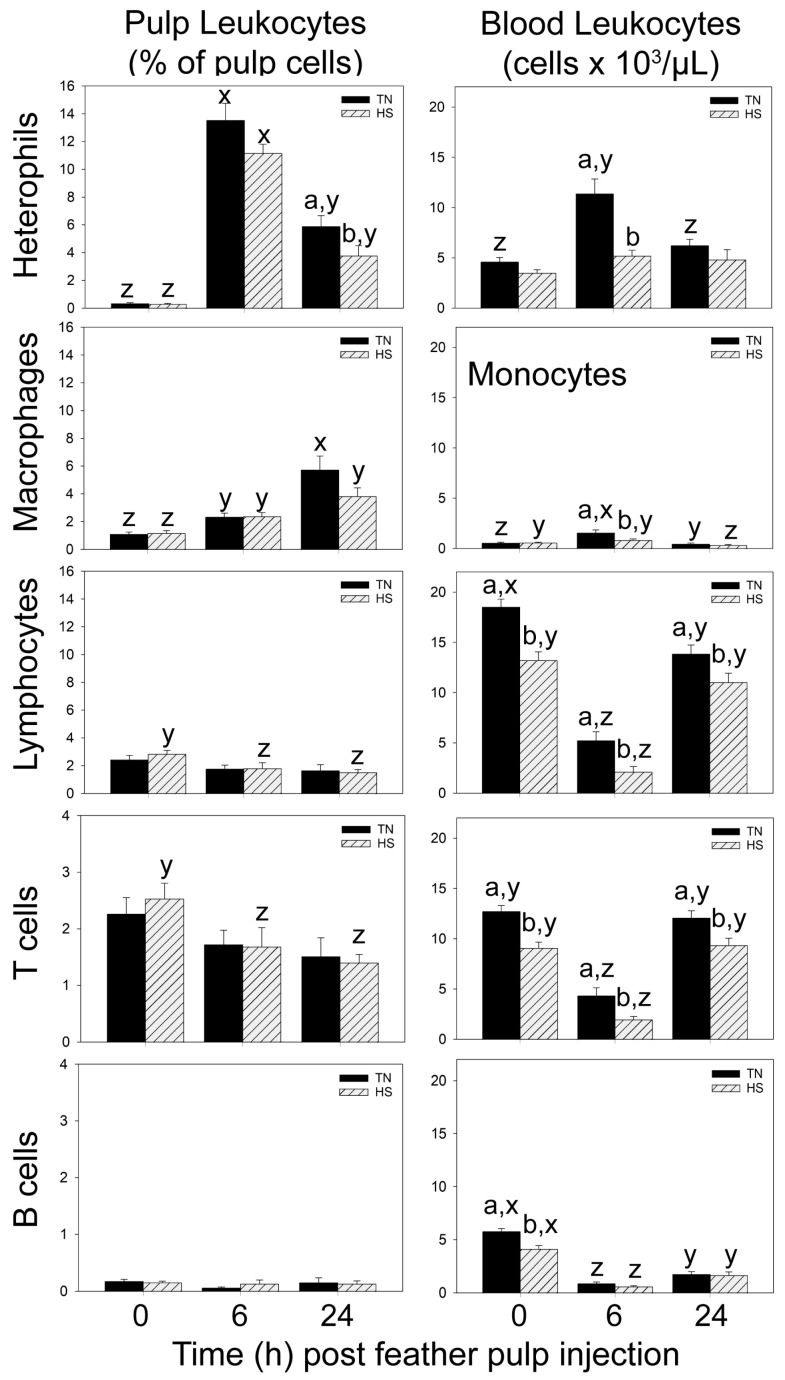
Leukocyte levels in growing feather (GF) pulps and blood before and after intradermal pulp injection of lipopolysaccharide (LPS) in 37-day-old broiler chickens reared in cyclic heat stress (HS) or thermal neutral (TN) conditions. Broilers were reared in TN- (Day 1–3, 32 °C; Day 4–6, 31 °C; Day 7–10, 29 °C; Day 11–14, 26 °C; Day 15 onward, 24 °C) or cyclic HS-conditions, i.e., 35 °C between 8:00 am and 10:00 pm and TN temperatures between 10:00 pm to 8:00 am, daily, from Day 4 onward. At 37 days of age, GF-pulps were injected with 10 μL of LPS (1 μg/GF, 12 GF/bird) and GF and blood collected before (0 h) and at 6 and 24 h post-injection. Pulp and whole blood cell suspensions were subjected to direct, 2–3 color, immunofluorescent staining using fluorescently labeled mouse monoclonal antibodies CD41/61-FITC (blood only), CD45-SPRD, KUL01-PE, CD3-PE and Bu-1-FITC to identify chicken thrombocytes, total leukocytes, monocytes/macrophages, T- and B cells, respectively. Cell population analysis was conducted by flow cytometry. Heterophils were identified within the CD45-postive population based on side- and forward-scatter characteristics [27]. Lymphocyte populations were calculated by addition of T and B cells. Data shown are mean leukocyte levels ± SEM; at 0 h, n = 12 broilers for TN/HS groups; at 6 and 24 h, n = 8 broilers for TN-/HS-LPS groups. a,b: means are different (*p* ≤ 0.05) between TN and HS within a time-point and cell type; y,z: within a cell-type and TN/HS group, means at different times without a common letter are different (*p* ≤ 0.05).

There were no differences (*p* > 0.05) in the levels of heterophils in pulps and blood between TN and HS broilers before (0 h) i.d. GF-pulp injections (Figure 1). For both HS- and TN-LPS broilers, levels (% pulp cells) of heterophils in the pulp increased greatly at 6 h post-injection (p.i.), then dropped at 24 h p.i., but remained above pre-injection levels. Pulp heterophil levels were lower in HS- than TN-LPS pulps at 24 h p.i. (Figure 1). In the blood, heterophil concentrations (cells/µL) were also elevated in TN-LPS broilers at 6 h, but returned to baseline levels at 24 h p.i. There was no change in heterophil concentrations in HS-LPS broilers in response to i.d. pulp injection of LPS. At 6 h p.i., heterophil concentrations in the blood of HS-LPS broilers were lower than in TN-LPS broilers (Figure 1).

Pulp injection of PBS (injection control) also resulted in elevated heterophil levels in GF-pulps of both TN- and HS-PBS broilers at 6 h p.i. (Table 1). Heterophil levels then dropped in pulps of TN-PBS broilers, but remained above pre-injection levels at 24 h p.i. in both TN- and HS-PBS birds. There were no differences in heterophil pulp-infiltration levels in TN- compared to HS-PBS broilers. The increase in heterophils at 6 h post-PBS injection was 7- to 9-fold lower than with LPS injection (Figure 1, Table 1). Heterophil concentrations in the blood did not change in response to i.d. GF-pulp injection of PBS Table 1).

#### 3.1.2. Macrophages/Monocytes

Prior to i.d. GF-pulp injection, there were no differences in macrophage levels in GF-pulps and monocyte concentrations in blood between TN and HS broilers (Figure 1). For both TN- and HS-LPS broilers, macrophage level in the pulp increased at 6 h p.i. Macrophage levels continued to increase in TN-LPS birds to highest levels at 24 h p.i., while no further increase was observed in HS-LPS pulps. There were no differences in macrophage levels between TN-LPS and HS-LPS broilers over the 24 h period (Figure 1). In the blood, monocyte concentrations were elevated in TN-LPS broilers at 6 h, whereas in HS-LPS broilers, monocyte concentrations remained unchanged at 6 h p.i. compared to 0 h and dropped slightly below baseline levels at 24 h p.i. Monocyte concentrations in HS-LPS broilers were lower than in TN-LPS broilers at 6 h p.i. (Figure 1).

Pulp injection of PBS (injection control) resulted in increased macrophage levels in pulps of TN-PBS broilers that reached highest levels at 24 h (Table 1). A similar but non-significant (*p* > 0.05) increase over time p.i. was also observed in HS-PBS broilers. The increase in pulp macrophage levels at 24 h post-PBS injection was approximately 3-fold lower than in response to LPS injection (Figure 1, Table 1). Monocyte concentrations in the blood did not change in response to i.d. GF-pulp injection of PBS.

#### 3.1.3. Lymphocytes

There were no differences in GF-pulp lymphocyte levels prior to i.d. injection, but lymphocyte levels were lower in HS-LPS birds at 6 h and 24 h p.i. than before (0 h) injection (Figure 1). This drop in GF-pulp lymphocytes of HS-LPS broilers was due to T cells as pulp B cell levels did not change over time. In TN-LPS broilers, neither total pulp lymphocyte levels nor the levels of T or B cell subpopulations changed post-LPS injection. However, blood concentrations of lymphocytes were lower in HS- than TN-broilers before (0 h) LPS injection, and at 6 and 24 h p.i. (Figure 1). In both TN- and HS-LPS broilers, blood lymphocyte concentrations dropped greatly at 6 h p.i. Blood lymphocyte concentrations increased again by 24 h, returning to baseline concentrations in HS-LPS, but remained below pre-injection concentrations in TN-LPS broilers. Interestingly, the lower concentrations of circulating lymphocytes in HS-broilers before GF-injection were due to lower concentrations of both T and B cells, while at 6 and 24 h p.i., the difference in blood lymphocyte concentrations was due to T cells only. Additionally, for both TN- and HS-LPS broilers, T cell concentrations returned to pre-injection levels at 24 h, while B cell concentrations remained below concentrations observed before i.d. LPS injection into GF-pulps (Figure 1).

Similar changes in GF and blood lymphocyte profiles were observed in response to i.d. GF-pulp injection of PBS (Table 1). In GF, lymphocyte levels dropped by 24 h p.i. to similar levels in both TN- and HS-PBS broilers. In pulps of TN-PBS broilers, this drop was due to both T and B cells, while in HS-PBS broilers, only a drop in T cell levels was observed. Moreover, B cell levels were higher in pulps of HS-PBS than in TN-PBS broilers at 6 and 24 h p.i. Blood lymphocyte concentration changes in response to PBS were similar to those observed with LPS, although the drop in total lymphocytes and, especially, T cells was comparatively smaller in TN-PBS vs. TN-LPS broilers, but not in HS-PBS vs. HS-LPS broilers (Figure 1, Table 1). The drop in circulating concentrations of B cells was similar in response to PBS and LPS in both HS and TN broilers. (Figure 1, Table 1).

#### 3.1.4. Other Blood Cell Populations and Heterophil to Lymphocyte Ratios

Before GF-pulp injections, there were no differences between TN- and HS-broilers in the blood concentrations of basophils, eosinophils, thrombocytes, and erythrocytes (Table 2). In both TN- and HS-broilers, GF-pulp injection with PBS did not alter the concentrations of these blood cells. Following GF-pulp injection with LPS, eosinophil and thrombocyte concentrations were lower and higher, respectively, at 6 h p.i., and returned to pre-injection concentrations by 24 h p.i. in both TN- and HS-broilers. Concentrations of basophils and erythrocytes were not affected by GF-pulp injection of LPS (Table 2). In HS-broilers, the H:L ratio was elevated at 6 h post-PBS and LPS injections, and returned to pre-injection levels at 24 h p.i. In TN-broilers, the H:L ratio did not change post-PBS injection, but was elevated at 6 h post-LPS injection and returned to pre-injection levels at 24 h p.i. The H:L ratio at 6 h post-PBS injection was higher in HS- than TN-broilers (Table 2).

### 3.2. Reactive Oxygen Species (ROS) Generation in GF-Pulps before and after i.d. GF-Pulp Injection of LPS or PBS

**Figure 2:**
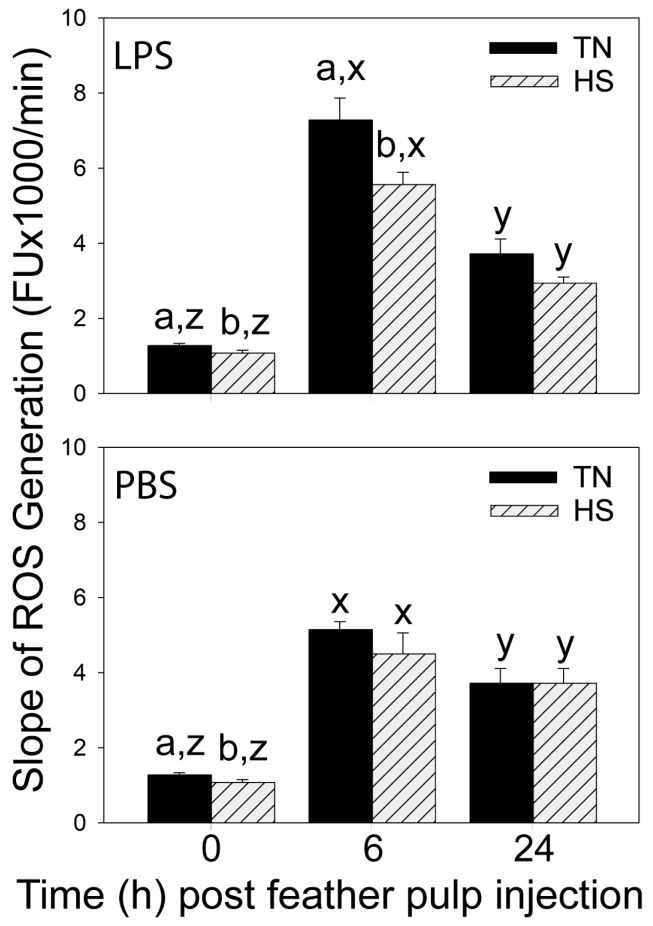
Reactive oxygen species (ROS) generation in growing feather (GF) pulps before and after intradermal pulp injection of lipopolysaccharide (LPS; top) or vehicle (PBS; bottom) in 37-day-old broiler chickens reared in cyclic heat stress (HS) or thermal neutral (TN) conditions. Broilers were reared in TN- (Day 1–3, 32 °C; Day 4–6, 31 °C; Day 7–10, 29 °C; Day 11–14, 26 °C; Day 15 onward, 24 °C) or cyclic HS-conditions, i.e., 35 °C between 8:00 am and 10:00 pm and TN temperatures between 10:00 pm to 8:00 am, daily, from Day 4 onward. At 37 days of age, 12 GF-pulps per bird were injected with 10 μL of LPS (1 μg/GF) or 10 µL endotoxin-free phosphate-buffered saline (PBS, vehicle). Pulp cell suspensions prepared from GFs collected before (0 h) and at 6 and 24 h post-GF injection were subjected to fluorescence kinetic assay and the generation of ROS expressed as the slope of fluorescence emission. Data shown are mean fluorescence units (FU) × 10^3^/min ± SEM; at 0 h, n = 12 broilers for TN/HS groups; at 6 and 24 h, n = 8 broilers for TN-/HS-LPS, 4 broilers for TN-/HS-PBS. For each LPS or PBS treatment, a,b: ROS generation means are different (*p* ≤ 0.05) between TN and HS within a time-point; y,z: within a TN/HS group, ROS generation means at different times without a common letter are different (*p* ≤ 0.05).

Prior to i.d. GF-pulp injections, ROS generation (slope; FU × 1000/min) in GF-pulps was higher in TN compared to HS broilers (Figure 2). The generation of ROS increased in TN- and HS-pulps in response to LPS injection, reaching highest levels at 6 h p.i. At 24 h p.i., ROS generation dropped in both TN- and HS-LPS broilers but remained above pre-injection levels. At 6 h p.i., ROS generation was lower in HS- compared to TN-LPS broilers (Figure 2).

In both TN- and HS-PBS broilers, ROS generation in GF-pulps also increased to highest levels by 6 h p.i., and dropped to above baseline levels by 24 h (Figure 2). At 6 h p.i., the peak ROS generation in response to PBS injection was approximately 2000 FU/min lower in TN-PBS than TN-LPS broilers, and only about 700 FU/min lower in HS-PBS than HS-LPS broilers (Figure 2).

### 3.3. Relative Cytokine mRNA Expression in GF-Pulps before and after i.d. GF-Pulp Injection of LPS or PBS

**Figure 3:**
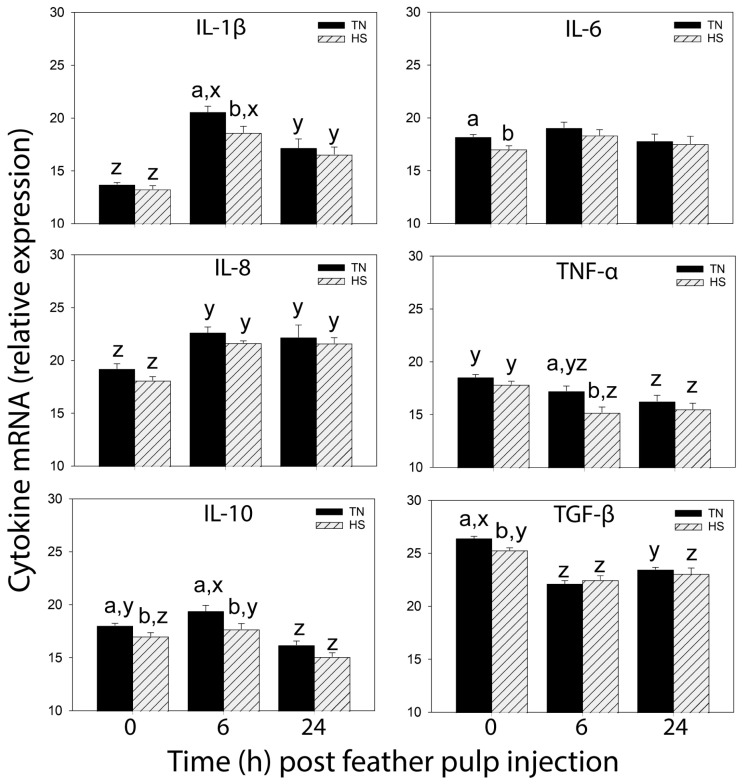
Relative cytokine mRNA expression levels in growing feather (GF) pulps before and after intradermal pulp injection of lipopolysaccharide (LPS) in 37-day-old broiler chickens reared in cyclic heat stress (HS) or thermal neutral (TN) conditions. Broilers were reared in TN- (Day 1–3, 32 °C; Day 4–6, 31 °C; Day 7–10, 29 °C; Day 11–14, 26 °C; Day 15 onward, 24 °C) or cyclic HS-conditions, i.e., 35 °C between 8:00 am and 10:00 pm and TN temperatures between 10:00 pm to 8:00 am, daily from Day 4 onward. At 37 days of age, GF-pulps were injected with 10 μL of LPS (1 μg/GF, 12 GF/bird) and GF collected before (0 h) and at 6 and 24 h post-injection for RNA isolation and relative gene-expression analysis by qRT-PCR of interleukin 1 (IL-1), IL-6, IL-8 (CXCL8), IL-10, tumor necrosis factor (TNF-α) and transforming growth factor-β1 (TGF-β1). Data shown are mean relative mRNA expression (40-delta Ct) ± SEM; at 0 h, n = 12 broilers for TN/HS groups; at 6 and 24 h, n = 8 broilers for TN-/HS-LPS groups. a,b: means are different (*p* ≤ 0.05) between TN and HS within a time-point and cytokine; y,z: within a cytokine and TN/HS group, means at different times without a common letter are different (*p* ≤ 0.05).

Prior to i.d. GF-pulp injections, relative mRNA expression levels (40-delta Ct) of IL-1β, IL-8, and TNF-α were not different in pulps of TN- and HS-broilers, while expression of IL-6, IL-10, and TGF-β1 was lower in GF-pulps of HS compared to TN broilers (Figure 3). In both TN- and HS-LPS broilers, mRNA expression levels of IL-1β, IL-8, and IL-10 were elevated at 6 h p.i. By 24 h, IL-1β mRNA expression dropped but remained above pre-injection levels, IL-8 expression remained similar to that at 6 h p.i., and IL-10 expression dropped to pre-injection levels in both TN- and HS-LPS broilers. In pulps of both TN- and HS-LPS broilers, IL-6 mRNA expression did not change over time, while TNF-α and TGF-β1 expression dropped post-LPS injection. Pre-injection differences in IL-6 and TGF-β1 mRNA expression between TN- and HS-broilers were no longer observed post-LPS injection. IL-10 and TNF-α mRNA expression was lower at 6 h p.i. in HS- than TN-LPS broilers (Figure 3).

In GF-pulps of both TN- and HS-PBS injection control birds, the relative expression of IL-1β and IL-8 mRNA was elevated at 6 and 24 h p.i. At 6 h, IL-1β mRNA expression was higher in TN-PBS compared to HS-PBS broilers (Table 3). The increases in IL-1β and IL-8 mRNA expression were comparatively higher in response to LPS vs. PBS injection (Figure 3; Table 3). mRNA expression of IL-6, IL-10, TNF-α, and TGF-β1 in GF-pulps did not change in response to i.d. injection of PBS in both TN- and HS-PBS broilers (Table 3).

### 3.4. Plasma Alpha-1-Acid Glycoprotein (AGP-1) Concentrations before and after i.d. GF-Pulp Injection of LPS or PBS

**Figure 4:**
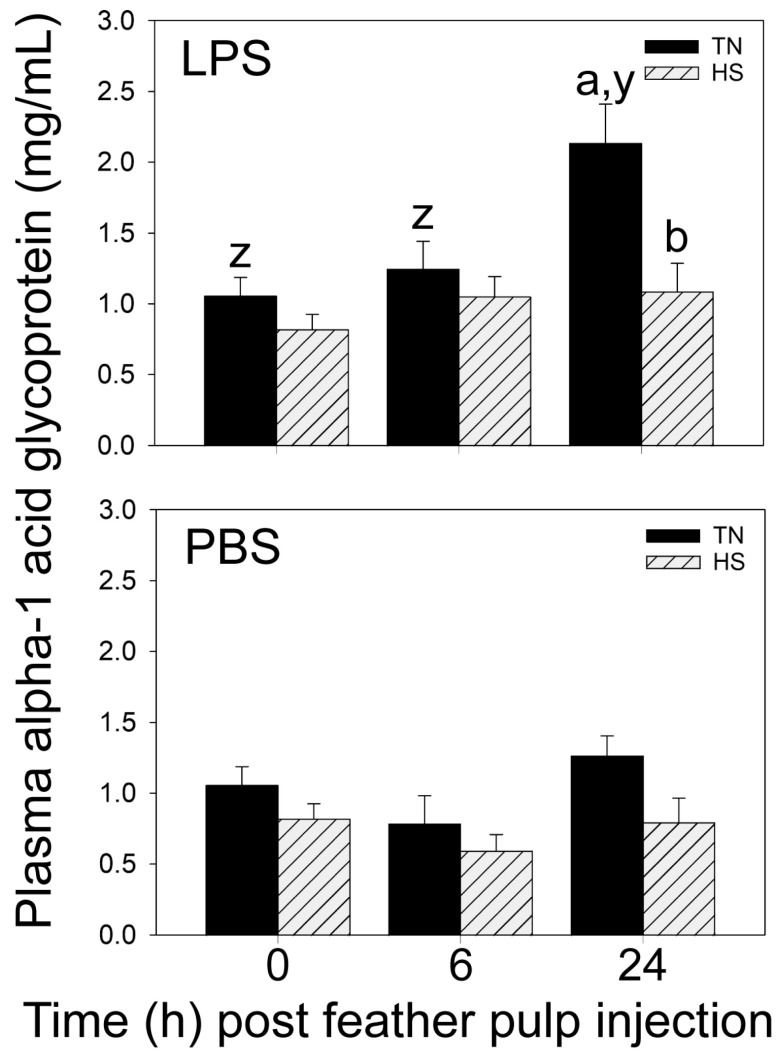
Plasma alpha-1 acid glycoprotein (AGP-1) concentrations in blood plasma before and after intradermal pulp injection of lipopolysaccharide (LPS) or vehicle (PBS) in 37-day-old broiler chickens reared in cyclic heat stress (HS) or thermal neutral (TN) conditions. Broilers were reared in TN- (Day 1–3, 32 °C; Day 4–6, 31 °C; Day 7–10, 29 °C; Day 11–14, 26 °C; Day 15 onward, 24 °C) or cyclic HS-conditions, i.e., 35 °C between 8:00 am and 10:00 pm and TN temperatures between 10:00 pm to 8:00 am, daily, from Day 4 onward. At 37 days of age, 12 GF-pulps per bird were injected with 10 μL of LPS (1 μg/GF) or 10 µL endotoxin-free phosphate-buffered saline (PBS, vehicle). Plasma from heparinized blood samples collected before (0 h) and at 6 and 24 h post-GF injection were subjected to AGP-1 ELISA. Data shown are mean AGP-1 concentrations (mg/mL) ± SEM; at 0 h, n = 12 broilers for TN/HS groups; at 6 and 24 h, n = 8 broilers for TN-/HS-LPS groups, 4 broilers for TN-/HS-PBS groups. For each LPS or PBS treatment, a,b: AGP-1 concentration means are different (*p* ≤ 0.05) between TN and HS within a time-point; y,z: within a TN/HS group, AGP-1 concentration means at different times without a common letter are different (*p* ≤ 0.05).

Prior to i.d. GF-pulp injections, there were no differences in AGP-1 concentrations (mg/mL) between TN and HS broilers (Figure 4). In both TN- and HS-LPS broilers, plasma AGP-1 concentrations were elevated by 24 h p.i., although AGP-1 concentrations were substantially higher in TN-LPS compared to HS-LPS broilers at this time-point (Figure 4).

In PBS injection controls, there was no change in plasma AGP-1 concentrations as a result of i.d. GP-pulp injection of PBS (Figure 4) in both TN- and HS-PBS broilers.

## 4. Discussion

Inflammation is a highly conserved, innate immune system response that acts as a major first line of defense in the fight against microbial infection. As shown by French et al. [23] using the “two window approach”, broilers exhibit all the basic hallmarks of LPS-stimulated inflammatory activities described [17,22]. Using a similar experimental approach as French et al. [23], we examined the effects of cyclic, environmental heat-stress on the local (GF-pulp) and systemic (blood) inflammatory responses initiated by i.d. GF-pulp injection of LPS. In the current study, observations in TN-broilers fully corroborated those reported by [23], further supporting the relevance of the insights gained from the two-window approach. Comparison of data from HS-broilers with those from TN-broilers revealed (1) suppressive effects of cyclic heat stress on various aspects of both the local and systemic inflammatory responses to LPS; (2) suppressive effects of cyclic heat stress on the response to sterile, endotoxin-free PBS injection (injection control); and (3), based on analysis of pre-injection samples, suppressive effects of cyclic heat stress on immune system development, including lower baseline ROS generation and mRNA expression of cytokines in GF-pulps (a skin derivative) and lower concentrations of circulating blood lymphocytes. To our knowledge, this is the first comprehensive assessment of the effects of environmental, cyclic heat stress on the local and systemic innate immune system activities in response to LPS and vehicle injection, as well as on aspects of baseline immune system status in broilers.

### 4.1. Suppressive Effects of Cyclic, Environmental Heat Stress on the Local and Systemic Responses to LPS Injected into the Dermis of GF-Pulps

In both HS- and TN-broilers, LPS administration stimulated the recruitment of heterophils and monocytes/macrophages, but not lymphocytes, from the blood into the injected GF-pulps. While heterophil and monocyte/macrophage infiltration was similar at 6 h p.i. in both HS- and TN-broilers, suppressive effects of heat stress on heterophil- and monocyte/macrophage-infiltration at the site of LPS injection were evident at 24 h. Specifically, at 24 h p.i., heterophil levels (% pulp cells) were lower in GF-pulps of HS- than TN-broilers, and in HS-broilers, there was no increase in macrophages from 6 to 24 h p.i. In both HS- and TN-broilers, ROS generation and relative mRNA expressions of IL-1β, IL-8, and IL-10 peaked at 6 h p.i. However, the 6 h peak in ROS generation and in relative mRNA expression of IL-1β and IL-10 were lower in GF-pulps of HS- than TN-broilers. Moreover, the drop in mRNA expression of TNF-α occurred earlier (6 h p.i.) in HS- than TN-broilers, and, although TGF-β1 mRNA expression dropped at 6 h p.i. in both HS- and TN-broilers, it only increased again by 24 h p.i. in TN-broilers. Taken together, the temporal and quantitative differences between the LPS-induced local inflammatory responses of HS- and TN-broilers attest to the suppressive effects of cyclic heat stress not only on leukocyte recruitment and infiltration, but also on the functional activities of resident/infiltrating leukocytes and/or other GF-pulp cells. Similar suppressive effects of heat stress on the inflammatory response were observed when broilers were subjected to heat stress and infected with Gram-negative bacteria (i.e., *Salmonella* Enteritidis [12], *Escherichia coli* [10]).

A reduced ability in HS-broilers to fully respond to the inflammatory activity initiated by LPS in the GF-pulp can also be observed in the peripheral blood. In the blood, the anticipated [23] increases in the heterophil and monocyte concentrations at 6 h post-GF-pulp injection of LPS only occurred in TN-broilers. In HS-broilers, the heterophil concentrations did not change post-LPS injection and the monocyte concentration gradually dropped below pre-injection levels at 24 h. These observations suggest a lower ability of HS-broilers to communicate the need for more heterophils and macrophages at the site of inflammation and/or to produce/release additional heterophils and monocytes into the circulation [17,30,31].

One effect of i.d. GF-pulp injection of LPS that was similar in magnitude in HS- and TN-broilers is the extensive drop in the concentrations of circulating lymphocytes at 6 h p.i. and their subsequent return to near baseline levels by 24 h p.i. In both HS- and TN-broilers, the 6 h drop in lymphocyte concentrations was due to reduced concentrations of T and B cells, with T cells returning to pre-injection concentrations at 24 h p.i., while B cells remained below pre-injection concentrations. The drop in blood lymphocyte concentrations in response to LPS administration has been consistently reported, but the mechanisms are not well understood and have primarily been attributed to the lymphotoxic effects of LPS [21,22,23,31]. However, as discussed below, a drop in blood lymphocyte concentrations was also observed in TN- and HS-broilers when sterile, endotoxin-free PBS (injection control) was injected into the dermis of GF-pulps, suggesting that the drop in circulating concentrations of lymphocytes post-LPS administration may in part be due to physiological effects associated with GF-pulp injection and handling of the broilers [23].

Other blood cell populations were also affected by i.d. GF-pulp injection of LPS. Included are a drop in eosinophil and an increase in thrombocyte concentrations at 6 h post-LPS injection in both TN- and HS-broilers. For TN-broilers, this observation differs somewhat from [23], which reported no change in eosinophils, and increased concentrations of thrombocytes at 24 h p.i. These discrepancies in eosinophil and thrombocytes concentrations changes in TN-broilers compared to French et al.’s [23] broilers may be due to the different methods employed to assess blood cell concentrations. Specifically, in the current study, a combination of immunofluorescence-based flow cytometry and Wright-stained blood smear evaluations were used to identify thrombocytes and eosinophils, respectively. In a previous study [23], proportions and concentrations of leukocytes were determined by automated hematology (i.e., Cell Dyn, Abbott Diagnostics, Abbott Park, IL, USA) calibrated for chicken blood cells.

Lastly, plasma AGP-1 concentrations increased to the highest levels in TN-broilers at 24 h after i.d. GF-pulp injection of LPS, whereas in HS-broilers, plasma AGP-1 concentrations did not change over time. Considering the protective and regulatory role of acute phase proteins such as AGP-1, this too is a negative effect of environmental HS on the innate immune system’s efforts to control inflammatory damage and restore homeostasis [17,32,33].

Collectively, our observations support suppressive effects of cyclic, environmental heat stress on the broilers’ ability to effectively respond to LPS administration and, hence, to challenges by Gram-negative bacteria.

### 4.2. Suppressive Effects of Cyclic Heat Stress on the Local and Systemic Inflammatory Responses to Injection of Sterile, Endotoxin-Free PBS into the Dermis of GF-Pulps

In both TN- and HS-broilers, few effects of the vehicle injection, namely sterile, endotoxin-free PBS (injection control), were observed both at the injection site and in the peripheral blood circulation. In PBS-injected GF, heterophil levels (% pulp cells) increased reaching peak levels at 6 h in both TN- and HS-broilers. However, while monocyte/macrophage infiltration was highest at 24 h p.i. in TN-broilers, it did not change from 6 to 24 h in HS-broilers. These responses in TN- and HS-broilers were similar to those observed with LPS, but the relative quantity of infiltrating cells was many folds lower with PBS than LPS. Furthermore, the small changes in heterophil and monocyte/macrophage presence in PBS-injected GF-pulps of TN-broilers are consistent with observations by French et al. [23]. The increases in local inflammatory activities such as ROS generation and the relative mRNA expression of proinflammatory cytokines IL-1β and IL-8, albeit lower than with LPS, further reflect local inflammatory activity initiated by injection of PBS due to tissue damage and activation of repair processes. Other than lower expression of IL-1β mRNA in HS- than TN-broilers at 6 h post i.d. PBS injection, there were no significant differences in local inflammatory activity due to cyclic heat stress. Like with LPS, lymphocytes were not recruited to GF-pulps in response to PBS injection. However, lower lymphocyte levels were observed at 24 h p.i. in GF-pulps of both HS- and TN-broilers, whereby this decrease was due to lower T cell presence. This small drop in GF-pulp lymphocytes observed here with the PBS-injections was not observed in [23], although they reported a drop in GF-pulp lymphocytes when LPS was injected. We do not have an explanation for these inconsistencies in GF-pulp lymphocyte levels, other than that the significance of these small proportional changes varies from experiment to experiment.

The increases in heterophils and monocytes/macrophages in GF-pulps post-PBS injection were not accompanied by changes in heterophil and monocyte concentrations in the blood circulation, in both HS- and TN-broilers. However, as mentioned above, a drop in blood lymphocyte concentrations was also observed in both HS- and TN-broilers in response to PBS injection. This observation suggests a contributing role of the stress associated with handling and injection to the drop in circulating lymphocyte concentrations seen with LPS [33]. It should be noted that the drop in circulating lymphocytes in TN-broilers was less extensive with PBS than with LPS, whereas in HS-broilers, the drop was almost identical in response to PBS or LPS. Hence, cyclic heat stress exacerbated this effect in HS-injection controls, suggesting a bigger role of physiological stress in the drop in lymphocyte concentrations in the HS-group. Moreover, in HS-broilers, the blood T cell concentrations dropped from 9.07 ± 0.60 × 10^3^ cells/µL before PBS injection to 3.07 ± 0.81 × 10^3^ cells/µL at 6 h p.i., whereas in TN-broilers, the drop was less extensive (i.e., from 12.7 ± 0.58 × 10^3^ cells/µL to 8.59 ± 0.98 × 10^3^ cells/µL). B cell concentrations dropped to a similar extent in HS- and TN-broilers; and, unlike T cells, which recovered to pre-injection concentrations at 24 h p.i., B cells remained at low concentrations at 24 h p.i. in both groups. Hence, the physiological stress associated with injections and handling of the broilers may affect the B cell compartment to a larger extent than T cells. The basis for this observation needs further investigation.

The exacerbating effect of cyclic heat stress on the reduction of circulating concentrations of lymphocytes in HS-broilers is further supported by the increase in H:L ratios at 6 h post-PBS-injection in HS- but not in TN-broilers. It is particularly notable that this increase in H:L ratio in HS-broilers was solely due to the drop in blood lymphocytes, as there was no increase in heterophil concentrations at this time-point.

The injection of sterile, endotoxin-free PBS into GF-pulps also did not affect the blood concentrations of basophils, eosinophils, thrombocytes, and erythrocytes. Moreover, i.d. injection of PBS without a microbial component did not stimulate an increase in plasma AGP-1 concentrations. This observation agrees with AGP-1′s role as an acute phase protein in cell protection and anti-inflammatory activities in response to microbial infection [17,32,33].

Taken together, few effects of the control injection and few differences between HS- and TN-broilers in the local and systemic inflammatory responses to PBS were observed. The extensive drop in blood lymphocytes in HS-broilers and significant increase in the H:L ratio at 6 h post-injection of sterile, endotoxin-free PBS suggests additional physiological stress in HS-broilers and an impaired ability of the HS-broilers to cope with the stress of being handled and with the tissue damage associated with the GF-pulp injection.

### 4.3. Suppressive Effects of Cyclic Heat Stress on Baseline Levels of Immune Cells and Activities in GF-Pulps and Blood

In GF-pulps, there were no differences in baseline levels (0 h) of heterophils, macrophages and lymphocytes, and in relative mRNA expression of pro-inflammatory cytokines IL-1β, IL-8 and TNF-α, between HS- and TN-broilers. Considering that the presence and production of inflammatory cells and cytokines are dependent on inflammatory stimuli, it is not surprising that baseline tissue levels are similar between HS- and TN-broilers. However, baseline ROS generation and relative expression of IL-6, IL-10, and TGF-β1 mRNA was lower in HS- compared to TN-broilers. These differences between HS- and TN-broilers may be due to suppressive effect of heat stress on metabolic activities of resident leukocytes and epidermal and dermal pulp cells [11,12,13].

It should be noted that, except for lymphocytes, the baseline levels of all other blood cells (i.e., RBC, thrombocytes, heterophils, monocytes, eosinophils, and basophils) were not different for broilers reared under HS- versus TN-conditions. Hence, it appears that cyclic heat stress alone did not affect hematopoiesis of myeloid cells. Rather, the lower concentrations of circulating T- and B-lymphocytes point towards an effect of cyclic heat stress on lymphocyte development in the thymus and bursa of Fabricius, respectively. In a similar study by Quinteiro-Filho et al. [10], 35-day-old broilers were subjected to 10 h of HS (36 °C), with control birds kept at TN (21 °C) temperatures for 24 h per day, for one week. At 42 days of age, HS broilers were reported to have elevated serum corticosterone concentrations, decreased relative weights (% BW) of the thymus and bursa of Fabricius, and reduced *Staphylococcus aureus*-induced reactive oxygen species generation (ROS) by macrophages in vitro. These alterations in the thymic and bursal weights, as well as ex vivo macrophage ROS generation, were attributed to the elevated levels of the stress hormone, corticosterone [10]. Corticosterone, as well as sex steroids, are known to drive regression of primary lymphoid organs by reducing the levels of immature lymphocytes (e.g., CD4^+^CD8^+^ thymocytes) and hence, the weight of these organs [17]. Moreover, corticosterone and other glucocorticoids are known to have anti-inflammatory properties, explaining the reduced ROS generation in response to *S. aureus* stimulation of macrophages in the Quinteiro-Filho et al. [10] study.

While corticosterone concentrations were not measured in the current study, our observations of reduced local and systemic acute inflammatory responses and reduced circulating levels of T- and B-lymphocytes in HS broilers are likely due to elevated levels of heat-stress-associated stress hormones. In avian species, the H:L ratio is often used as an indicator of physiological stress [31,34,35]. With this assumption, the similarly low H:L ratios before PBS/LPS injection suggests that environmental, cyclic heat stress did not impose an additional stress on the broilers. This may be due to habituation of HS-broilers to the heat over the 33 days of high temperatures from 8:00 am to 10 pm. On the other hand, the 6 h increase in H:L ratio following PBS-injection in HS- but not in TN-broilers may be interpreted as an indicator of increased physiological stress in HS-broilers, as there was no microbial stimulus or non-sterile injury influencing the H:L ratio. The great increase in H:L ratio at 6 h post-LPS injection in both HS- and TN-broilers, however, is primarily due to the inflammatory response to LPS and, hence, “immunological stress”. This observation serves as a reminder that in order to use the H:L ratio as an indicator of physiological stress, microbial infection status of the chicken has to be considered. Based on the current study, it appears that increased H:L ratios during an inflammatory response in TN-broilers involve both an increase in blood heterophil concentrations and a drop in the concentrations of blood lymphocytes, while changes in H:L ratios due to physiological stress may be due to a drop in lymphocyte concentrations only.

## 5. Conclusions

The observed suppression of LPS-stimulated, local and systemic acute inflammatory responses and in circulating levels of lymphocytes in HS broilers, suggest that cyclic, environmental heat stress conditions may impair the ability of broilers to mount effective innate immune responses, which may prove to be problematic for maintaining flock health as HS continues to grow as an issue. Further research should be performed to elucidate the mechanisms and extent of this impaired immune function in high temperature conditions. Application of the dual-window approach used here could prove to be useful in selecting individuals exhibiting greater immune robustness while experiencing heat stress.

## Figures and Tables

**Table 1:** Leukocyte profiles in growing feather (GF) pulps and blood following intradermal pulp injection of endotoxin-free phosphate-buffered saline (PBS) vehicle in 37-day-old broiler chickens reared in cyclic heat stress or thermal neutral conditions.

Growing Feather Pulp (% Pulp Cells)					Blood (Cells × 10^3^/µL)		
Leukocyte	Trt ^1^	0 h ^2^	6 h	24 h	0 h	6 h	24 h
Heterophil	TN	0.36 ± 0.03 z	1.98 ± 0.70 x	0.91 ± 0.34 y	4.61 ± 0.43	5.20 ± 0.63	5.30 ± 0.78
	HS	0.32 ± 0.03 z	1.37 ± 0.22 y	1.04 ± 0.30 y	3.52 ± 0.31	3.98 ± 0.86	5.80 ± 0.99
Macro/Mono ^3^	TN	1.12 ± 0.13 z	1.42 ± 0.26 yz	2.29 ± 1.01 y	0.56 ± 0.06	0.81 ± 0.08	0.42 ± 0.08
	HS	1.18 ± 0.17	1.27 ± 0.12	1.73 ± 0.60	0.58 ± 0.05	1.01 ± 0.44	0.51 ± 0.05
Lymphocyte	TN	2.44 ± 0.30 y	2.73 ± 0.50 y	1.35 ± 0.43 z	18.5 ± 0.78 a,y	9.87 ± 1.29 a,z	15.3 ± 1.15 y
	HS	2.96 ± 0.24 y	3.13 ± 0.41 y	1.54 ± 0.19 z	13.2 ± 0.87 b,y	3.73 ± 0.90 b,z	10.5 ± 1.36 y
T cell	TN	2.26 ± 0.29 y	2.65 ± 0.48 y	1.27 ± 0.41 z	12.7 ± 0.58 a,y	8.59 ± 0.98 a,z	13.7 ± 0.99 a,y
	HS	2.53 ± 0.28 yz	2.96 ± 0.37 y	1.94 ± 0.37 z	9.07 ± 0.60 b,y	3.07 ± 0.81 b,z	9.09 ± 1.16 b,y
B cell	TN	0.18 ± 0.03 y	0.08 ± 0.03 b,z	0.08 ± 0.02 b,z	5.80 ± 0.26 a,y	1.28 ± 0.31 z	1.59 ± 0.29 z
	HS	0.16 ± 0.02	0.18 ± 0.06 a	0.17 ± 0.04 a	4.13 ± 0.32 b,y	0.66 ± 0.06 z	1.38 ± 0.25 z

^1^ Trt = temperature treatment; TN = thermoneutral rearing conditions, i.e., Day 1–3, 32 °C; Day 4–6, 31 °C; Day 7–10, 29 °C; Day 11–14, 26 °C; Day 15 onward, 24 °C; HS = cyclic heat stress conditions, i.e., from Day 4 onward, 35 °C 8:00 am–10:00 pm, TN temperatures 10:00 pm–8:00 am. ^2^ Time (h) with respect to PBS injection into GF pulps; i.e., before (0 h), or 6 or 24 h post-injection; ^3^ Macro/mono = macrophages/monocytes; GF-pulps were injected with 10 μL of PBS. GF and heparinized blood were collected before (0 h) and at 6 and 24 h post-GF injection. GF-pulp and whole blood cell suspensions were subjected to direct, 2–3 color, immunofluorescent staining using fluorescently labeled mouse monoclonal antibodies CD41/61-FITC (blood only), CD45-SPRD, KUL01-PE, CD3-PE and Bu-1-FITC to identify chicken thrombocytes, total leukocytes, monocytes/macrophages, T and B cells, respectively. Cell population analysis was conducted by flow cytometry. Heterophils were identified within the CD45-postive population based on side- and forward-scatter characteristics [27]. Lymphocyte populations were calculated by addition of T and B cells. Data shown are mean ± SEM; at 0 h, n = 12 broilers for TN/HS groups; at 6 and 24 h, n = 4 broilers for TN-/HS-PBS groups. a,b: within a column and cell type, means are different (*p* ≤ 0.05); y,z: within a tissue (GF-pulp or blood), cell type, and Trt group, means at different times without a common letter are different (*p* ≤ 0.05).

**Table 2:** Leukocyte profiles in blood following intradermal pulp injection of endotoxin-free phosphate-buffered saline (PBS) vehicle or lipopolysaccharide (LPS) in 37-day-old broiler chickens reared in cyclic heat stress or thermal neutral conditions.

		Pre-Injection	Post-PBS-Injection		Post-LPS Injection	
Cell Type/Ratio	Trt ^1^	0 h	6 h	24 h	6 h	24 h
Basophils × 10^3^/µL	TN	0.500 ± 0.072	0.518 ± 0.090	0.612 ± 0.264	0.429 ± 0.078	0.647 ± 0.206
	HS	0.324 ± 0.047	0.362 ± 0.134	0.468 ± 0.222	0.159 ± 0.036	0.422 ± 0.143
Eosinophils × 10^3^/µL	TN	0.536 ± 0.065	0.424 ± 0.124	0.608 ± 0.152	0.223 ± 0.055 *	0.549 ± 0.068
	HS	0.416 ± 0.049	0.279 ± 0.049	0.397 ± 0.167	0.122 ± 0.023 *	0.387 ± 0.070
Thrombocytes × 10^3^/µL	TN	8.19 ± 1.16	10.3 ± 1.21	13.2 ± 3.01	13.4 ± 1.15 *	9.66 ± 1.01
	HS	5.99 ± 0.69	9.83 ± 1.40	7.34 ± 1.38	9.66 ± 0.57 *	6.85 ± 0.76
Erythrocytes × 10^6^/µL	TN	2.79 ± 0.16	2.79 ± 0.32	2.80 ± 0.05	2.49 ± 0.20	2.82 ± 1.01
	HS	2.56 ± 0.10	2.22 ± 0.12	2.46 ± 0.09	2.08 ± 0.11	2.35 ± 0.06
H:L Ratio ^2^	TN	0.25 ± 0.02	0.53 ± 0.04 b	0.36 ± 0.07	2.57 ± 0.06 *	0.45 ± 0.04
	HS	0.28 ± 0.03	1.22 ± 0.39 a *	0.57 ± 0.04	2.27 ± 0.24 *	0.43 ± 0.07

^1^ Trt = temperature treatment; TN = thermoneutral rearing conditions, i.e., Day 1–3, 32 °C; Day 4–6, 31 °C; Day 7–10, 29 °C; Day 11–14, 26 °C; Day 15 onward, 24 °C; HS = cyclic heat-stress conditions, i.e., from Day 4 onward, 35 °C 8:00 am–10:00 pm, TN temperatures 10:00 pm–8:00 am. ^2^ H:L Ratio = heterophil to lymphocyte ratio. GF-pulps were injected with 10 μL of PBS/LPS (1 μg LPS/GF). Heparinized blood was collected before (0 h) and at 6 and 24 h post-GF injection. Whole blood cell suspensions were subjected to direct, 2-color, immunofluorescent staining using fluorescently labeled mouse monoclonal antibodies CD41/61-FITC, CD45-SPRD, to identify chicken thrombocytes and total leukocytes. Cell population analysis was conducted by flow cytometry. The proportions of basophils and eosinophils were determined based on evaluation of at least 300 leukocytes on a Wright stained blood smear by microscopy (1000× magnification). The concentration of basophils and eosinophils was calculated based on the concentration of WBC (CD45+CD41/61−) determined by flow cytometry. Data shown are mean ± SEM; at 0 h, n = 12 broilers for TN/HS groups; at 6 and 24 h, n = 8 broilers for TN- and n = 4 broilers for HA-groups. *: For each cell type/ratio and within a Trt and injection group, mean is different from pre-injection mean (*p* ≤ 0.05); a,b: Within a cell type/ratio and time-point, Trt means are different.

**Table 3:** Relative cytokine mRNA expression levels in growing feather (GF) pulps following intradermal pulp injection of endotoxin-free phosphate-buffered saline (PBS) diluent in 37-day-old broiler chickens reared in cyclic heat stress or thermal neutral conditions.

Cytokine mRNA (Relative Expression)				
Cytokine	Trt ^1^	0 h ^2^	6 h	24 h
IL-1β	TN	13.7 ± 0.17 z	16.7 ± 0.79 a,y	16.0 ± 0.52 y
	HS	13.3 ± 0.31 z	14.6 ± 0.40 b,yz	15.9 ± 0.17 y
IL-6	TN	18.2 ± 0.22	18.0 ± 0.57	18.5 ± 0.54
	HS	17.0 ± 0.31	17.6 ± 0.42	18.3 ± 0.58
IL-8 (CXCL8)	TN	19.2 ± 0.46 a,z	20.4 ± 0.69 yz	21.7 ± 0.40 y
	HS	18.1 ± 0.33 b,z	20.2 ± 0.52 y	21.7 ± 0.39 y
TNF-α	TN	18.5 ± 0.25	18.1 ± 0.56	18.2 ± 0.25
	HS	17.9 ± 0.29	17.3 ± 0.49	17.4 ± 0.32
IL-10	TN	18.0 ± 0.20 a	17.6 ± 0.64	17.7 ± 0.37
	HS	17.0 ± 0.32 b	16.6 ± 0.12	16.7 ± 0.25
TGF-β1	TN	26.4 ± 0.18 a	25.5 ± 0.54	25.4 ± 0.53
	HS	25.3 ± 0.22 b	24.8 ± 0.25	24.9 ± 0.54

^1^ Trt = temperature treatment; TN = thermoneutral rearing conditions, i.e., Day 1–3, 32 °C; Day 4–6, 31 °C; Day 7–10, 29 °C; Day 11–14, 26 °C; Day 15 onward, 24 °C; HS = cyclic heat-stress conditions, i.e., from Day 4 onward, 35 °C 8:00 am–10:00 pm, TN temperatures 10:00 pm–8:00 am. ^2^ Time (h) with respect to PBS injection into GF pulps; i.e., before (0 h), or 6 and 24 h post-injection; GF-pulps were injected with 10 μL of PBS. Pulp RNA isolated from GFs collected before (0 h) and at 6 and 24 h post-GF injection was subjected to qRT-PCR analysis. Data shown are mean relative expression (40-delta CT) ± SEM; at 0 h, n = 12 broilers for TN/HS groups; at 6 and 24 h, n = 4 broilers for TN-/HS-PBS groups. a,b: within a column and cytokine, means are different (*p* ≤ 0.05); y,z: within a cytokine and Trt group, means at different times without a common letter are different (*p* ≤ 0.05).

## Data Availability

Data are available upon request from the corresponding author.
